# Effects of Oxygen–Ozone Injections in Upper Limb Disorders: Scoping Review

**DOI:** 10.3390/jcm14072452

**Published:** 2025-04-03

**Authors:** Gianpaolo Ronconi, Ariani Mariantonietta, Sefora Codazza, Alberto Cutaia, Alessandra Zeni, Lucia Forastiere, Giorgio Ferriero, Paola Emilia Ferrara

**Affiliations:** 1 Department of Geriatrics, Orthopedics and Rheumatology, Università Cattolica del Sacro Cuore, L.go F. Vito 1, 00168 Rome, Italy; gianpaolo.ronconi@policlincogemelli.it; 2 Fondazione Policlinico Universitario “Agostino Gemelli” IRCCS, Department of Geriatrics, Orthopedics and Rheumatology, L.go A. Gemelli 8, 00168 Rome, Italy; paolaemiliaferrara@policlinicogemelli.it; 3 ASL Frosinone, Complex Operational Unit, Integrated Home Care Assistance, 00163 Rome, Italy; sefora.codazza@conv.aslfrosinone.it; 4 Department of Neurosciens, Sense Organs and Thorax, Catholic University of the Sacred Heart, 00168 Rome, Italy; alberto.cutaia@guest.policlinicogemelli.it (A.C.); alessandra.zeni@guest.policlinicogemelli.it (A.Z.);; 5 Unit of Physical and Rehabilitation Medicine, Istituti Clinici Scientifici Maugeri IRCCS, 21049 Tradate, Italy; giorgio.ferriero@icsmaugeri.it; 6 Department of Biotechnology and Life Sciences, University of Insubria, 21110 Varese, Italy

**Keywords:** corticosteroid injection, elbow, hand, intra-articular injections, outcome, oxygen–ozone therapy, rehabilitation, shoulder, upper limb, wrist

## Abstract

**Background:** Ozone therapy is used for its immunomodulatory, antioxidant, and analgesic properties in several fields. It can be useful in the rehabilitation of musculoskeletal disorders. Studies showed that O_2_-O_3_ therapy can reduce pain and improve functioning in patients affected by low back pain and knee osteoarthritis. Only a few studies have been published about the efficacy of this treatment in upper limb disease. **Objective**: The aim of this study is to investigate the use of ozone therapy in upper limb pathologies, evaluating its quantity, quality, and reported results in upper limb musculoskeletal disease, supraspinatus tendinopathy, shoulder impingement, adhesive capsulitis, chronic epicondylitis, and carpal tunnel syndrome. O_2_-O_3_ reduces inflammation by stimulating anti-inflammatory cytokines and inactivating pro-inflammatory molecules, relieves pain by interacting with pain receptors and improving blood circulation, promotes the regeneration of damaged tissues by stimulating growth factors and improving vascularization, and, finally, activates endogenous antioxidant defense systems by protecting cells from oxidative damage. **Methods**: A comprehensive search was conducted on PubMed and Scopus using the following MeSH terms: ozone therapy, infiltration joint, musculoskeletal disease, rehabilitation, upper limb, shoulder, wrist, hand, elbow, including English papers published in the last five years. **Results**: Five papers have been selected: four randomized controlled trials and one retrospective cohort study. The RCTs compared the effectiveness of intra-articular ozone injection with steroid injection alone or with other conservative treatments in shoulder diseases; one paper studied the effectiveness of ozone injection and orthoses in carpal tunnel syndrome compared to orthoses alone; one paper used ozone injections compared with steroid injection in patients with chronic lateral epicondylitis. A total of 218 patients were studied in these trials. **Conclusions**: Ozone treatment seemed to improve pain and function as well as other therapies in upper limb musculoskeletal disease. However, the trials’ protocols and the upper limb areas treated are different. Further studies are needed to define the effectiveness of ozone therapy in upper limb diseases in rehabilitation fields.

## 1. Introduction

Ozone therapy (O_2_-O_3_) has been used as a treatment for muscle, tendon, and joint disorders. Several studies have evaluated the effectiveness of O_2_-O_3_ therapy in musculoskeletal disorders, including low back pain, lumbar disc herniation, failed back syndrome, degenerative spine disease, plantar fasciitis, and knee osteoarthritis [1]. It is injected peri-articularly, intra-articularly, or percutaneously [2].

For example, intra-articular ozone injection has a good result in pain management in knee osteoarthritis (KOA) and it can be a potential treatment due to its safety and ease of administration. Although there are differences in the results, possibly due to the different protocols used in the treatment of knee osteoarthritis regarding both ozone concentration and the number of injections performed, ozone therapy appears to be a promising area of research for treating KOA [3].

O_2_-O_3_ therapy is commonly used as an adjunctive treatment for various conditions characterized by chronic inflammation and immune system overactivation, including musculoskeletal disorders [4]. Disorders in the upper limb include rotator cuff tendinopathies, lateral elbow epicondylitis, carpal tunnel syndrome, frozen shoulder, and osteoarthritis, leading to pain and functional limitation.

Ozone (O_3_) is an inorganic molecule with allotropic properties consisting of three oxygen atoms. Ozone is an unstable molecule that cannot be stored. It is generated by a device that converts pure O_2_ into O_3_ by passing it through a high-voltage gradient (5–13 mmV) [4]. The actions of ozone include: bactericidal and virucidal, anti-inflammatory and immunomodulatory, antioxidant, and analgesic actions.

Like any other therapeutic intervention, O_2_-O_3_ therapy has contraindications, that include: glucose-6-phosphate dehydrogenase deficiency, pregnancy, uncontrolled hyperthyroidism, severe cardiovascular disease, and heart failure ^4^. The concentration of ozone used in different pathologies varies depending on the type of pathology and the site to be treated.

The upper limb, like other parts of the body, can be affected by various musculoskeletal pathologies. To date, the use of O_2_-O_3_ molecules mainly occurs in pathologies of the lower limb and in pathologies concerning the spine. The aim of this study is to investigate the use of ozone therapy in upper limb pathologies, evaluating its quantity, quality, and reported results in upper limb musculoskeletal disease.

## 2. Materials and Methods

**Figure 1:**
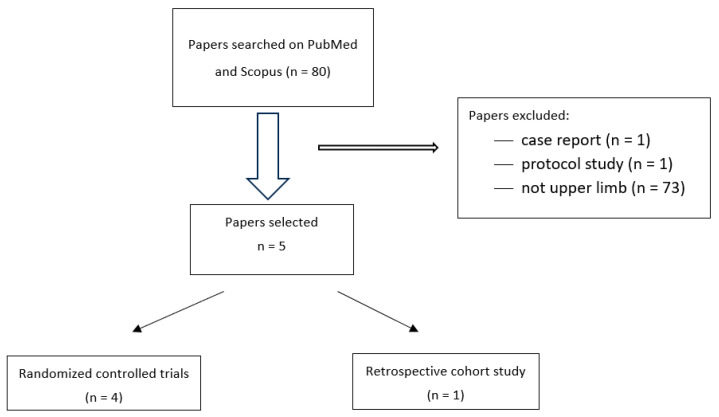
Study selection process for intra-articular ozone injections.

We performed a search on PubMed and Scopus (Figure 1). We used the following MeSH terms: “tendinitis” AND “ozone”, “ozone” AND “intra-articular injection”, “ozone” AND “intra-articular injection shoulder”, “ozone” AND “intra-articular injection” AND “elbow”, “ozone” AND “intra-articular injection” AND “hand”, “ozone” AND “shoulder pain”, “injection therapy” AND “ozone therapy”.

Our research yielded a selection of articles published between 2019 and 2023.

Inclusion criteria were reporting randomized controlled trials (RCTs), retrospective cohort studies, and including adults (aged 18 years or older) with osteoarticular and musculoskeletal disorders of the upper limb. Exclusion criteria were case reports and protocol studies.

The articles were independently reviewed by multiple reviewers. All articles were either excluded or included based on the full-text review. No reviewer had any conflicts of interest.

**Figure 2:**
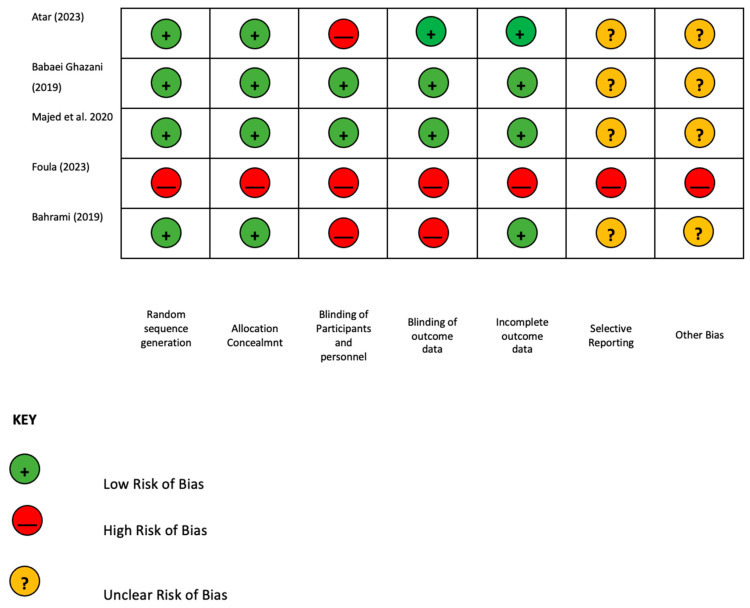
Risk of bias [1,6,7,8,9].

The methodology of this study was reported following the Cochrane Library assessment tool used to evaluate the risk of bias in the studies selected [5]. The Cochrane tool includes seven domains (sequence generation, allocation concealment, blinding of participants and personnel, blinding of outcome data, incomplete outcome data, selective outcome reporting, and other sources of bias). A green light is assigned to a low risk of bias, a yellow light to an unclear risk of bias, and a red light to a high risk of bias (Figure 2).

## 3. Results

The literature search identified 80 studies but only 7 studies deal with the treatment of osteoarticular and musculoskeletal disorders of the upper limb using ozone compared to other treatment modalities. We included five articles: four randomized controlled trials (RCTs) and one retrospective study (Figure 1).

The included articles are research papers published between 2019 and 2023. Of these, four are randomized controlled trials designed to assess the effectiveness of specific interventions. One article is a cohort study, which follows a group of individuals over time to investigate specific outcomes. There is no pooled analysis, meta-analysis, heterogeneity assessment, or evaluation of publication bias.

The Cochrane Library assessment tool was used to evaluate the risk of bias for all the RCT studies included [1,6,7,8]. Two studies presented a low risk of bias [1,7]; Atar et al. and Bahrami et al. showed a high risk of bias in blinding participants, personnel, and outcome data; Ulusoy et al. was a retrospective study without any RCT criteria (Figure 2).

Three studies focused on treating patients with various shoulder conditions, totaling 119 participants. The conditions included adhesive capsulitis, chronic tendon disorders, and shoulder impingement. A majority of these patients were female. The remaining two studies examined different areas: tennis elbow (lateral epicondylitis) and carpal tunnel syndrome.

The number of patients in each study was relatively small. In the study that discussed shoulder impingement [1], the effects of oxygen–ozone injections in 15 patients with shoulder impingement were compared to a similarly sized control group. All groups performed the same physical therapy: shoulder ROM exercises, posterior capsule stretching, and isometric exercises. The study on adhesive capsulitis [7] also investigated oxygen–ozone injections in 15 patients, contrasting them with two control groups of equal size that received different treatments. The study on lateral epicondylitis [9] was the largest, evaluating the outcomes of oxygen–ozone therapy in 42 patients and comparing them to a control group of 38 patients.

In these studies, oxygen–ozone therapy was compared to use of corticosteroids and intra-articular radiofrequency. The treatment of different pathologies with oxygen–ozone infiltration varied in terms of both gas concentration and the number of infiltrations. The minimum number of infiltrations (one session) was performed in three studies [1,7,8], while the maximum number was six to eight injections. Concentrations also varied across studies (from 10 mcg/mL to 30 mcg/mL) [9]. Even in control groups, the type and dosage of corticosteroid interventions varied. In adhesive capsulitis [7], two control groups were used: one received corticosteroid infiltration and the other received intra-articular platelet-rich plasma (PRP). In carpal tunnel syndrome [8], one control group received wrist orthosis. Outcome measures included the Visual Analog Scale (VAS) for pain assessment in all studies, except for lateral epicondylitis [9], where the Verhaar criteria scale was used to evaluate pain at rest, during compression, and during movement. Studies on shoulder pathologies [1,6,7] also evaluated the Shoulder Pain and Disability Index (SPADI) and the Western Ontario and McMaster Universities Arthritis Index (WORC) to assess shoulder disability and range of motion. Additionally, in adhesive capsulitis [7], biomarkers of inflammation were evaluated. In the study of carpal tunnel syndrome [8], in addition to VAS, the Brief Pain Inventory (BPI) and nerve conduction velocity (NCV) parameters were used to assess symptom severity, functionality, and nerve function. Follow-up periods in shoulder studies ranged up to 12 weeks [6], while patients in the lateral epicondylitis study [9] were evaluated for up to 9 months.

In studies of shoulder pathologies [1,6,7], improvements in pain, quality of life, and function were similar between the study and control groups. In the study of adhesive capsulitis [7], shoulder disability improved similarly across groups, with a more significant decrease in VAS at 8 weeks in the PRP group who used a NeuroTherm^®^ NT2000iX RF machine with a 10 mm active tip (NeuroTherm, manufactured by Abbott (Chicago U.S.A.) for 4 min. ROM improved more at follow-up in the PRP group, followed by the ozone group and then the steroid group. Biomarkers of inflammation improved in the second and fourth follow-up weeks. In the shoulder impingement study, corticosteroid infiltration was more effective than ozone infiltration. In the study of chronic lateral epicondylitis, Verhaar criteria improved at follow-up in the ozone group compared to the steroid group. In carpal tunnel syndrome [8], VAS and BQ outcomes improved in patients treated with ozone and orthosis (prefabricated wrist-based resting splint with a metal bar on the volar side for eight weeks put on during the night and for most waking hours) compared to those treated with orthosis alone.

## 4. Discussion

Oxygen–ozone (O_2_-O_3_) therapy has been studied for over a century. Recent studies have suggested local ozone injection as a therapeutic option for musculoskeletal conditions such as low back pain, knee osteoarthritis, cervical pain, tendinopathies, and fibromyalgia [4,8]. This review aims to evaluate the effectiveness of ozone injections compared to other treatments for upper limb musculoskeletal disorders. Our review identified five studies, including four randomized controlled trials (RCTs) and one cohort study, investigating the use of ozone therapy in musculoskeletal and tendon pathologies of the upper limb, as detailed in Table 1. Three studies evaluated the use of ozone in shoulder pathologies [1,6,7], employing different protocols while assessing the same outcomes (WORC, SPADI, VAS). The study of chronic supraspinatus tendon pathology [6] showed no significant difference between the group treated with corticosteroid infiltration and the group treated with three oxygen–ozone infiltrations. Sun Y. et al. [10] demonstrated that intra-articular steroid injection is effective and safe for frozen shoulder, relieving pain, improving functional performance, and increasing ROM.

The study of adhesive capsulitis [6] in 45 patients compared three treatment types: oxygen–ozone infiltration, corticosteroid injection, and PRP. Results showed that shoulder disability improved similarly across all groups. However, the Visual Analog Scale (VAS) demonstrated a significantly greater reduction at 8 weeks in the PRP group. Additionally, the range of motion (ROM) improved more significantly in the PRP group, followed by the ozone treatment group, with the steroid control group showing the least improvement. Furthermore, inflammatory biomarkers showed positive changes in both the second and fourth follow-up weeks.

The study on shoulder impingement [1] compared corticosteroid injections with ozone injections, showing clear benefits for corticosteroid infiltration. Corticosteroid injections in the subacromial region significantly improved pain and functional status in patients with subacromial impingement syndrome during short-term follow-up [11]. Moreover, as already studied for hip injections, the association between steroid and hyaluronic acid in patients with hip osteoarthritis requires more rigorous studies with a larger sample size to define the best practices [12]. Additionally, Say et al. [13] found improvements in Constant score and VAS pain scores at 6 and 12 weeks in patients who received a single-dose steroid injection compared to a single-dose PRP injection.

Ulusoy et al. [9] demonstrated that 42 patients receiving 6–8 oxygen–ozone injections every three days experienced improved pain at rest and during movement in the third, sixth, and ninth weeks compared to 38 patients who received a cycle of injections with betamethasone dipropionate and betamethasone sodium phosphate once a week for 3 weeks. The conservative treatment of this condition has been investigated in various studies. Among the injectable therapies, corticosteroids, analgesics, platelet-rich plasma, and autologous whole blood have been studied [14]. Corticosteroid injections are commonly used and have shown effectiveness in reducing pain and enhancing functional outcomes in the short and medium term. However, long-term use may result in adverse effects, including tendon weakening and limited pain control over time [14]. A meta-analysis [15] found that autologous blood injections were more effective in alleviating pain compared to corticosteroid injections in lateral epicondylosis, so may serve as a viable alternative treatment option. Additionally, it was found that autologous blood and PRP showed no significant differences in the treatment of this condition. Furthermore, Kemp JA et al. [16] demonstrated that PRP is a superior long-term treatment option for lateral epicondylosis compared to corticosteroid injections, while corticosteroid injections have been shown to provide short-term pain relief. PRP has also been shown to be effective in other pathologies, such as hip osteoarthritis, providing short-term pain relief and long-term functional improvement [17].

The study addressing carpal tunnel syndrome (grades 2 or 3) [8] demonstrated that ozone infiltration followed by a prefabricated wrist-based device improved outcomes (VAS, BQ, median NCS) compared to using the device alone. However, as reported by Ashworth et al. [18], there is no clear consensus on the best therapeutic choice between local corticosteroid injection and surgery for carpal tunnel syndrome. Our data suggest that oxygen–ozone could be a viable alternative for treating upper limb pathologies, given the few known contraindications [18], although a standardized treatment protocol for each pathology is lacking. Additionally, even treatments combining corticosteroids, instrumental physical therapies like PRP, and anesthetics have shown to be equally effective, with improvements in ROM and pain exceeding those of infiltrative therapy [7].

While some studies in the literature report short- to medium-term efficacy of corticosteroid injections in treating lateral epicondylitis, oxygen–ozone therapy appears to be an effective treatment option [9]. Further research is needed to establish a standardized ozone treatment protocol. Carpal tunnel syndrome often requires invasive surgical treatment; however, as demonstrated by Baharami et al. [8], oxygen–ozone therapy could be a viable conservative treatment option. Additional studies are needed to further explore this potential.

### Limits

This review has some limitations. Except for two trials [1,7], almost all the studies presented multiple biases in terms of blinding of participants and personnel and blinding of the outcome data. Another limitation is the small sample size across all studies and the variation in upper limb pathologies examined. So, we can describe only preliminary data about ozone therapy in upper limb disease.

## 5. Conclusions

Ozone therapy is progressively gaining recognition as a potential therapeutic option for various musculoskeletal pathologies, including those affecting the upper limb. Studies conducted thus far, and evaluated by us, have demonstrated promising results regarding its effectiveness in reducing pain and improving functionality in diverse conditions, such as tendinopathies, osteoarthritis, and compressive syndromes.

In particular, several investigations suggest that the injection of ozone directly into the affected tissues may exert an anti-inflammatory and analgesic effect, comparable to other conventional treatments. However, it is important to emphasize that, despite these encouraging results, the majority of studies present certain methodological limitations, such as a reduced sample size or a lack of adequate control groups.

Furthermore, there is a degree of heterogeneity in the treatment protocols utilized across different studies, particularly with regard to ozone concentration and the number of sessions required. This variability renders it challenging to standardize a unique therapeutic approach and definitively validate the efficacy of ozone therapy.

Notwithstanding these challenges, ozone therapy has demonstrated safety and good tolerability in the majority of patients, with rare side effects and limited contraindications. Its ease of administration renders it a potentially advantageous therapeutic option in diverse pathologies of the upper limb.

However, it is crucial to underscore that scientific research in this field is still in its early stages and necessitates further investigation. High-quality, multicenter studies, with a larger patient sample and standardized treatment protocols, are necessary to confirm the benefits of ozone therapy and define the optimal therapeutic strategies for the various pathologies of the upper limb.

## Figures and Tables

**Table 1:** Characteristics of the studies.

Author (Years)	Design of the Study	Pathology	Study Group	Control Group	Control Group	Outcome Mesaures	Follow-Up	Results
Atar 2022 [6]	Randomized clinical trial	Chronic supraspinatus tendinopathy	Three sessions (1 session/week) of 5 mL of ozone (O_2_-O_3_) (with a concentration of 10 μg/mL in the first session, 15 μg/ mL in the second session, and 20 μg/mL in the third session) N patient: 20 M/F: 6/14 Mean age (SD): 48. (10.38)	A mixture of 1 mL corticosteroid (betamethasone 3 mg/mL) and 1 mL lidocaine (20 mg) N patient: 20 M/F: 11/9 Mean age (SD): 50.15 (12.75)	-	WORC, SPADI, VAS	T0: baseline T1: 4 weeks T3: weeks	No differences between groups until T3 in pain quality of life and function.
Babaei-Ghazani 2019 [1]	Double-blind randomized controlled trial	Shoulder impingement	1 injection of 8 mL of ozone (O_2_-O_3_) with a concentration of 12 μg/mL and 2 mL of lidocaine 1%, plus 20 min local cold pack, plus physical therapy N patients: 15 M/F: 2/13 Mean age (SD): 59.40 (10.31)	1 injection of a mixture of 1 mL of triamcinolone 40 mg/mL with 2 mL of lidocaine 1%, plus 20 min local cold pack, plus physical therapy N patients: 15 M/F: 6/9 Mean age (SD): 58.80 (14.72)	-	VAS, Constant score, SPADI	T0: Postinjection T1: 2 weeks T2: 2 months	Corticosteroid injection improves (VAS *p* level 0.109, SPADI *p* level 0.295) the pain and disability scores more significantly than a one-time ozone injection.
Foula 2023 [7]	Double-blind randomized controlled trial	Shoulder adhesive capsulitis	Intra-articular injection of 10 mL of an oxygen–ozone mixture (15 μg/mL), plus 5 mL of 0.125% bupivacaine N patients: 15 M/F: 3/12 Mean age: 48 (SD)	Intra-articular injection of 40 mg triamcinolone, plus 5 mL of 0.125% bupivacaine N patients: 15 M/F: 3/12 Mean age: 42 (SD)	One session of radiofrequency (PRP), plus intra-articular injection of 5 mL of 0.125% bupivacaine PRP application N patients: 15 M/F: 7/8 Mean age: 46 (SD)	VAS during movement (VASm) and during rest (VASr), ROM, SPADI, inflammatory biomarkers (ICAM 1, hs-CPR)	T0: Preinjection T1: 2 h posttherapy T2: 1° week T3: 2° week T4: 4° week T5: 8° week	No differences between groups in VASm and VASr until T4. Significantly better scores at T5 in the PRP group. Significant improvement in VAS in “within analysis”. After T2 better results in VASr in steroid treatment group. Statistically significant ROM improvement in PRP group until T5. No differences between groups in SPADI. No differences in all groups in ICAM and hs-CRP at T0 and T4. Within analysis showed statistically significant (*p* level 0.001) improvement in both ICAM-1 and hs-CRP in all groups.
Ulusoy et al. 2019 [9]	Retrospective cohort study	Chronic lateral epicondylitis	6–8 injections with 3-day intervals, aliquots of 3 mL O_2_-O_3_) (concentration of 30 μg/mL) N patients: 42 M/F: 11/31 Mean age (SD): 45.1 (8.1)	1 injection every 3 weeks: 1 mL of betamethasone dipropionate (6.43 mg) and betamethasone sodium phosphate (2.63 mg) N patients: 38 M/F: 13/25 Mean age (SD): 46.4 (6.8)	-	Pain at rest. Pain on compression and during activity. All scores examined by modified Verhaar criteria	T0: After the injection of corticosteroid or ozone T1: 3 months after injection T2: 6 months after injection T3: 9 months after injection	At T0 and T1 there was no difference between corticosteroid and ozone groups with respect to pain. Analysis of pain at T1, T2, T3 demonstrated that ozone group had significantly better scores (*p* level < 0.001).
Bahrami et al. 2019 [8]	Randomized controlled trial	Mild or moderate carpal tunnel syndrome (CTS)	Wrist resting splint + a single local injection of 4 mL ozone (10 micrograms/dl) plus 1 mL lidocaine (1%) N patients: 18 M/F: 0/18 Mean age (SD): 48.27 (3.33)	Wrist resting splint: eight weeks N patients: 20 M/F: 0/18 Mean age (SD): 46.35 (6.3)	-	VAS, BQ symptom severity (BQ-SSS), functional status (BQ-FSS), median NCS (CMAP and SNAP)	T0: Baseline T1: 10 weeks	Significant improvement in study group after T1 in pain (VAS *p* level 0.42), BQ-SSS (*p* level 0.62), BQ-FSS (0.30) No differences in median NCS in groups.

## Data Availability

As reported in the Materials and Methods, the analyzed data were found by performing a search on PubMed and Scopus.
